# Capturing and missing the patient's story through outcome measures: A thematic comparison of patient‐generated items in PSYCHLOPS with CORE‐OM and PHQ‐9

**DOI:** 10.1111/hex.12652

**Published:** 2017-11-22

**Authors:** Célia MD Sales, Inês TD Neves, Paula G. Alves, Mark Ashworth

**Affiliations:** ^1^ Faculty of Psychology and Education Science at the University of Porto (FPCEUP) Center for Psychology at the University of Porto (CPUP) University of Porto Porto Portugal; ^2^ Faculty of Medicine Institute of Preventive Medicine and Public Health University of Lisbon Lisboa Portugal; ^3^ Instituto Universitário de Lisboa (ISCTE) Cis‐IUL Lisboa Portugal; ^4^ Research Department of Primary Care and Population Health University College London London UK; ^5^ Department of Primary Care and Public Health Sciences King's College London London UK

**Keywords:** individualised PROMS, outcome assessment, patient‐centred outome, patient‐generated measures, thematic analysis

## Abstract

**Background:**

There is increasing interest in individualized patient‐reported outcome measures (I‐PROMS), where patients themselves indicate the specific problems they want to address in therapy and these problems are used as items within the outcome measurement tool.

**Objective:**

This paper examined the extent to which 279 items reported in an I‐PROM (PSYCHLOPS) added qualitative information which was not captured by two well‐established outcome measures (CORE‐OM and PHQ‐9).

**Design:**

Comparison of items was only conducted for patients scoring above the “caseness” threshold on the standardized measures.

**Setting and patients:**

107 patients were participating in therapy within addiction and general psychiatric clinical settings.

**Main results:**

Almost every patient (95%) reported at least one item whose content was not covered by PHQ‐9, and 71% reported at least one item not covered by CORE‐OM.

**Discussion:**

Results demonstrate the relevance of individualized outcome assessment for capturing data describing the issues of greatest concern to patients, as nomothetic measures do not always seem to capture the whole story.

## 1 INTRODUCTION

Recent studies have shown a renewed interest in the individualized assessment of change during talking therapies.^1^, ^2^, ^3^ The strategy relies on the use of individualized patient‐reported outcome measures (I‐PROMS), where patients themselves indicate the specific problems they want to address in therapy and these problems are used as items within the measurement tool. It is assumed that such an individualized approach is more able to capture the uniqueness of each patient's condition. However, there is scant evidence supporting this assumption. In this study, we contrast an I‐PROM with two well‐established standardized outcome tools, in order to identify the extent to which patients add items that are not covered by standardized tools.

Psychological outcome assessment typically uses repeated administration of standardized self‐report instruments (PROMS), in order to detect the patient change over time according to nomothetic principles of classical psychometrics. The same instrument, measuring a construct with fixed pre‐selected items that capture variance on universal dimensions of the construct, is administered to all people within a defined population. Assessment judgements about one individual are based on comparison with other persons (clinical and non‐clinical populations) answering the same standardized items. However, by being designed with reference to the whole population, nomothetic PROMS probably under‐report individual‐specific problems presented by patients, and may contain items of little personal significance.^4^, ^5^, ^6^, ^7^ Extensive research and clinical practice have shown that there are significant between‐person differences in behaviour disorders (eg Diagnostic and Statistical Manual of Mental Disorders, DSM‐5),^8^ which cannot be captured by the nomothetic approach. For example, to assess treatment outcomes for depression, disorder‐specific PROMS may be used, which will locate each patient score relative to population norms, thus allowing the formulation of a formal diagnose with acceptable levels of between‐diagnostician agreement. Inferences based on these scores can be made about clinical recovery by comparison with dysfunctional population scoring levels, as well as informing epidemiological and evidence‐practice research (eg identifying effective treatments for depression). However, nomothetic PROMS may not identify and measure change on key variables that afflict individual depressed patients, including his or her specific context, difficulties and treatment priorities. To measure treatment outcomes without missing the uniqueness of the patient's condition requires an idiographic assessment approach, that is using psychological assessment instruments tailored for each individual.^2^, ^9^ Individualized PROMS^2,^ also called patient‐generated outcome measures, evaluate the degree to which a patient changes on items selected by the patient. Items correspond to personally defined problems or pertinent situational variables that can serve as indicators of change on aspects of importance to each patient.^2^, ^10^

One advantage of I‐PROMS is that increased attention is given to patients’ preferences and wishes in relation to their health care, which is more aligned with the values of patient‐centred care.^11^ Also, therapists claim that the routine use of I‐PROMS is beneficial in preparation for clinical sessions, to elaborate discussions after completion of the session, for supervision meetings, and for making clinical decisions concerning treatment.^12^, ^13^ Furthermore, there is evidence that I‐PROMS show greater sensitivity to change than nomothetic measures, as the evaluation of change is based on the problems directly elicited by patients,^4^, ^14^ and they present acceptable psychometric properties.^1^ Despite these advantages, we still know little about what we gain using I‐PROMS: if we ask patients what their problems are, to what extent will they report items that are not covered at all by well‐established PROMS? To our knowledge, the only previous study to address this question compared one of the most commonly used I‐PROMS, PSYCHLOPS (Psychological Outcome Profile’ see http://www.psychlops.org.uk), with CORE‐OM in a community‐based talking therapy service in primary care.^5^ The results showed that 60% of patients provided novel and relevant clinical information in their free‐text responses that would otherwise not have been considered in their outcome assessment.^5^ Our study aims to expand on these findings by extending the comparison of PSYCHLOPS to include both CORE‐OM, a general measure of psychological distress and PHQ‐9, another nomothetic measure in widespread use but with narrow depression‐specific focus, and to test these instruments in an entirely different population.

## 2 METHODS

### 2.1 Participants

Two distinct samples (psychiatric patients and drug and alcohol patients, total n = 107) were enrolled, in order to provide a broad clinical range and ensure that findings would be generalizable to a secondary care population. The inclusion criteria were to be over 18 years of age and undergoing outpatient treatment. Samples were recruited as part of a larger research programme, the International network for Personalising Health Assessment (IPHA).^3^ For the first sample, a total of 57 psychiatric patients were recruited from the psychiatric department of a general hospital serving the Alentejo area (Portugal). Two were excluded because of incomplete data collection leaving a final sample of 55 patients. A further 52 patients were recruited from three institutions specializing in the treatment of drug and alcohol addiction disorders in the Lisbon area (Portugal) (see Table S1).

### 2.2 Instruments

*PSYCHLOPS* 15 is a brief I‐PROM containing three free‐text items indicated by the patient: “Choose the problems that troubles you most,” “Choose another problem that troubles you” and “Choose one thing that is hard to do because of your problem (or problems).” Each free‐text item is scored on a 6‐point Likert scale for severity (from “0 =  not at all affected”, to “5 =  severely affected”) and duration (from “0 =  under 1 month”, to “5 =  over 5 years”). PSYCHLOPS also contains a fourth preset question (“How have you felt in yourself this last week,” scored from “0 =  very good” to “5 =  very bad”). These questions cover three domains: problems, functioning and well‐being although we excluded well‐being as this question was standardized and contained no qualitative data. The final comparison between instruments was conducted using PSYCHLOPS data obtained from responses to the problem and functioning domains.

*Clinical Outcomes in Routine Evaluation—Outcome Measure* 16 is a 34‐item self‐report measure consisting of 4 dimensions: well‐being (four items); social functioning (twelve items); problems/symptoms (twelve items); and risk (six items). Each item is rated on a 5‐point Likert scale ranging from 0 = not at all, to 4 = most or all the time, referring to patient experience over the last week.

*Patient Health Questionnaire—9 items* 17 is a 9‐item multipurpose tool for screening, diagnosing and monitoring the severity of depression according to DSM‐IV‐R criteria. Items are scored from 0 (“not at all”) to 3 (“nearly every day”). Questions refer to patient experience over the preceding fortnight. All measures were administered in Portuguese.

### 2.3 Procedure

Patients were invited to arrive at the hospital one hour prior to their first appointment, for a pre‐treatment evaluation session. PSYCHLOPS was the first instrument to be administered, followed by CORE‐OM and PHQ‐9 in random order; a socio‐demographic data collection form was presented at the end. Patients with literacy or visual problems were not excluded but offered support by a research assistant who administered the tools orally. The analysis procedure followed three major steps:

#### 2.3.1 Free‐text coding

The free‐text responses were coded using a 61 subtheme classification system,^18^ previously used by Ashworth et al^5^ for comparing PSYCHLOPS and CORE‐OM. If a response did not clearly fit into an existing subtheme, a new subtheme was created. Four new subthemes were added, resulting in a categorization system of 65 codes. Despite respondents being asked to list one problem per question, some listed more than one, in which case only the first problem mentioned was analysed.^5^ Validity was ensured by three independent judges (IN, RC and CB) coding each item independently, and when agreement could not be reached, a process of triangulation was adopted in discussion with the study supervisor (CS). Inter‐rater reliability, given by the average of Cohen's kappa across all rater pairs, was strong (problem 1—0, 81; problem 2—0,83; functionality item—0,89). Triangulation was required in less than 1% of the items (n = 14).

#### 2.3.2 Content matching

The 65 subthemes derived from PSYCHLOPS responses were compared with the content of CORE‐OM and PHQ‐9 (see Tables S2 and S3, respectively). Two independent judges (IN and RC) determined whether each subtheme did or did not map directly to items included in CORE‐OM and PHQ‐9, classifying the matching into one of four categories: (1) *definite yes*:** t**here is a direct and clear matching of contents (eg subtheme “Sleeping problems” and CORE‐OM/PHQ‐9 item that reports problems in sleeping); (2) *possible yes*: subtheme reports a problem that is probably related to a problem reported on CORE‐OM or PHQ‐9 (eg problems of “concentration at work” could probably be connected to CORE‐OM/PHQ‐9 anxiety items); (3) p*ossible no:* vague subthemes, or general, that might or might not be associated with CORE‐OM or PHQ‐9 items (eg “Relationships” is a vague statement and difficult to determine whether it is matched to any CORE‐OM or PHQ‐9 item); (4) *definite no:* clearly there is no matching, subtheme with a different content. When agreement could not be reached, a third judge was consulted and the original free‐text responses on PSYCHLOPS were compared with the CORE‐OM (or PHQ‐9) items to provide more evidence on matching. Inter‐rater reliability (two‐way mixed intraclass correlations, average‐measures, absolute agreement) was strong for content matching with CORE‐OM (ranging from 0.92 in item 23 to 1.00) and PHQ‐9 (ranging from 0.99 in item 9 to 1.00). Judges were aware of the aim of the categorization (they knew that hypothetically I‐PROMS capture additional information in the outcome measurement process), which could result in coding bias. To minimize this effect, a separate coding database was prepared for each coder containing the free‐text items only, that is anonymized and without information concerning patients’ demographic or clinical data, as well as scorings on PSYCHLOPS or in the nomothetic counterparts. Moreover, content non‐matching was only categorized for “*definite no”* items; items classified as “*possible no*” were not categorized as non‐matching.

#### 2.3.3 Descriptive statistics

We calculated the frequency of each subtheme found in PSYCHLOPS and the frequency of patients who indicated each subtheme in PSYCHLOPS. We also calculated the numbers and proportion of patients above the clinical threshold who indicated at least one subtheme which did not map into CORE‐OM and PHQ‐9 (ie frequency of patients with at least one “*definite no”* item). This comparison was confined to patients who were classified as “cases” by each nomothetic instrument. As such, we compared PSYCHLOPS with PHQ‐9 only for items formulated by patients classified as depressed and PSYCHLOPS with CORE‐OM only for patients classified as having clinical psychological distress. For CORE‐OM, “caseness” was defined as patients with a score of ≥10 and for PHQ‐9 a score of ≥10.^19^, ^20^ IBM SPSS Statistics 21^®^ was used.

## 3 RESULTS

The 279 items indicated by patients in PSYCHLOPS were classified into 51 subthemes out of a possible maximum of 65 subthemes. The most frequent contents were work‐related problems (26%) (eg “*losing my job at the end of the contract”*) and relational problems within the family, namely, being worried about someone in the family (23% of the subthemes). In all, just over half of the subthemes described some type of relational difficulties, such as breaking up with a partnership (eg “*I have no courage to break up with my husband”*), or relational difficulties within the family (eg “*knowing that my mother is alone with my father at home”*). Patients in both samples indicated problems which contained a large range of themes (sample 1‐ 42 themes; sample 2‐35 themes). As shown in Table S4, patients entering substance misuse treatment reported more addiction, work‐related and money problems, whereas patients in psychiatric setting indicated more often being worried about someone in their family and worries about health. The comparison between the 51 PSYCHLOPS subthemes and the two nomothetic measures showed that a large proportion of subthemes were not present in CORE‐OM (33.3% classified as “*definite no”*) nor in PHQ‐9 (84.3%, “*definite no”*) (see Table S4). A large proportion of patients within the clinical range reported at least one subtheme in PSYCHLOPS that was not covered by CORE‐OM or by PHQ‐9: 71% and 95%, respectively (Tables S4 and S5). This pattern of responses was observed in both samples (summary in Table S5).

## 4 DISCUSSION

These results add to earlier findings by Ashworth et al^5^ and confirm that PSYCHLOPS adds novel information to CORE‐OM as well as to PHQ‐9. The finding that two of the most widespread outcome measures do not capture the problems experienced by a large proportion of patients entering talking therapy challenges conventional approaches to conducting outcome assessment in routine clinical settings. The most common assessment strategy is to combine generic measures, such as CORE‐OM, which allow comparison of cases and services (key inputs for health quality management), with population‐specific measures that have a narrower focus and cover particular aspects associated with specific health impairment conditions, such as PHQ‐9. However, our results show that the range of problems people report is more diverse. Adding measurement tools to the assessment protocol in order to elicit a wider range of problems may not be a realistic strategy, especially in routine clinical settings, where feasibility and acceptability issues call for simple outcome assessment protocols and short measures. Brief individualized measures (PSYCHLOPS is a one‐page measure designed for self‐completion) may offer a valuable strategy for personalizing assessment, by giving patients the opportunity to tailor the evaluation process, and having “a legitimate voice in informing the items that determine the status of their outcomes”.^21^ Implications of our results for outcome assessment concern the significance of themes declared on I‐PROMS but not on standardized measures which will require further research. A key issue is the extent to which these uncaptured themes are relevant to therapy progress or outcome of therapy. It may be the case that they covary with themes present in the standardized inventories, thus having little impact on the measurement of change over the course of the treatment. We do not know whether captured or uncaptured themes demonstrate greater responsiveness to change in I‐PROMS. Despite these limits, we have demonstrated that a substantial proportion of reported psychological distress is not captured by two of the most widely used and validated mental health outcome measures. Does this mean we are missing the patient's story? Our preliminary findings support the use of I‐PROMS to capture a more complete version of the patient's story.

## References

[1] Elliott R , Wagner J , Sales CMD , Rodgers B , Alves P , Café M . Psychometrics of the personal questionnaire: a client‐generated outcome measure. Psychol Assess. 2016;28:263‐278. 26075406 10.1037/pas0000174

[2] Sales CMD , Alves P . Patient centred assessment in Psychotherapy: a review of individualised tools. Clin Psychol Sci Pract. 2016;23:265‐283.

[3] Sales CMD , Alves P , Evans C , Elliott R , On behalf of IPHA Group . The Individualised Patient‐Progress System (IPPS): a decade of international collaborative networking. Couns Psychother Res 2014;14:181‐191.

[4] Ashworth M , Evans C , Clement S . Measuring psychological outcomes after cognitive behavior therapy in primary care: a comparison between a new patient‐generated measure “PSYCHLOPS” (Psychological Outcome Profiles) and “HADS” (Hospital Anxiety and Depression Scale). J Ment Health. 2009;18:1‐9.

[5] Ashworth M , Robinson S , Evans C , Shepherd M , Conolly A , Rowlands G . What does an idiographic measure (PSYCHLOPS) tell us about the spectrum of psychological issues and scores on a nomothetic measure (CORE‐OM)? Prim Care Community Psychiatry. 2007;12:7‐16.

[6] Hédinsson H , Kristjánsdóttir H , Ólason D , Sigurosson J . A validation and replication study of the patient‐generated measure PSYCHLOPS on an Icelandic Clinical Population. Eur J Psychol Assess. 2013;29:89‐95.

[7] Sales CMD , Alves P . Individualized patient‐progress systems: why we need to move towards a personalized evaluation of psychological treatments. Can Psychol. 2012;53:115‐121.

[8] American Psychiatric Association . Diagnostic and Statistical Manual of Mental Disorders, 5th edn Washington, DC: American Psychiatric Publishing; 2013.

[9] Crawford MJ , Rutter D , Manley C , et al. Systematic review of involving patients in the planning and development of health care. BMJ. 2002;325:1‐5. 12458240 10.1136/bmj.325.7375.1263 PMC136920

[10] Haynes SN , Mumma GM , Pinson C . Idiographic assessment: conceptual and psychometric foundations of individualized behavioral assessment. Clin Psychol Rev. 2009;29:179‐191. 19217703 10.1016/j.cpr.2008.12.003

[11] Fitzpatrick R , Davey C , Buxton MJ , Jones DR . Evaluating patient‐based outcome measures for use in clinical trials. Health Technol Assess. 1998;2:1‐86. 9812244

[12] Ashworth M , Robinson S , Godfrey E , et al. The experiences of therapists using a new client‐centred psychometric instrument, ‘PSYCHLOPS’ (‘Psychological Outcome Profiles’). Couns Psychother Res. 2005;5:37‐41.

[13] Sales CMD , Gonçalves S , Fragoeiro A , Noronha S , Elliott R . Psychotherapists openness to routine naturalistic idiographic research? Ment Health Learn Disabil Res Pract. 2007;4:145‐161.

[14] Ashworth M , Robinson S , Godfrey E , et al. Measuring mental health outcomes in primary care: the psychometric properties of a new patient‐generated outcome measure, “PSYCHLOPS” (psychological outcome profiles). Prim Care Ment Health. 2005;3:261‐270.

[15] Ashworth M , Shepherd M , Chistey J , et al. A client‐generated psychometric instrument: the development of “PSYCHLOPS”. Couns Psychother Res. 2004;4:27‐31.

[16] Evans C , Mellor‐Clark J , Margison F , et al. CORE: clinical outcomes in routine evaluation. J Ment Health. 2000;9:247‐255.

[17] Kroenke K , Spitzer R . The PHQ‐9: a new depression diagnostic and severity measure. Psychiatr Ann. 2002;32:1‐7.

[18] Robinson S , Ashworth M , Shepherd M , Evans C . In their own words: a narrative‐based classification of clients’ problems on an idiographic outcome measure for talking therapy in primary care. Prim Care Ment Health 2006;4:165‐173.

[19] Connell J , Barkham M , Stiles WB , et al. Distribution of CORE‐OM scores in a general population, clinical cut‐off points and comparison with the CIS‐R. Br J Psychiatry. 2007;190:69‐74. 17197659 10.1192/bjp.bp.105.017657

[20] Improving Access to Psychological Treatments (IAPT). The IAPT Data Handbook: Guidance on recording and monitoring outcomes to support local evidence‐based practice (version 2.0): National Health Services (NHS) 2012.

[21] Barkham M . Patient‐centered assessment in psychotherapy: toward a greater bandwidth of evidence. Clin Psychol Sci Pract. 2016;23:284‐287.

