# Supplementary material for: Capturing and missing the patient's story through outcome measures: A thematic comparison of patient‐generated items in PSYCHLOPS with CORE‐OM and PHQ‐9
Source: Health Expect. 2017 Nov 22;21(3):615–9. doi: 10.1111/hex.12652 (PMC5980523; doi:10.1111/hex.12652)
Supplement: Supplementary file 1 [file HEX-21-615-s001.docx]

SUPPLEMENTARY Table 1. Sociodemographic characteristics of sample 1 and sample 2.

|  | Sample 1 (n=55) | |  | Sample 2 (n=52) | |
| --- | --- | --- | --- | --- | --- |
| Variable | n (%) | Mean (SD) |  | n (%) | Mean (SD) |
| Age (in years) |  | 42.6 (15.4) |  |  | 40.0 (11.7) |
| Gender (female) | 42 (76.4) |  |  | 14 (26.9) |  |
| Education level |  |  |  |  |  |
| Illiterate | 1 (1.8) |  |  | 0 (0.0) |  |
| Up to 4^th^ year of education | 9 (16.4) |  |  | 9 (17.3) |  |
| 5^th^ to 6^th^ year of education | 10 (18.2) |  |  | 8 (15.4) |  |
| 7^th^ to 9^th^ year of education | 15 (27.3) |  |  | 13 (25.0) |  |
| 10^th^ to 12^th^ year of education | 12 (21.8) |  |  | 7 (13.5) |  |
| University attendance | 5 (9.1) |  |  | 4 (7.7) |  |
| BSc/Msc/PhD | 3 (5.5) |  |  | 1 (1.9) |  |
| Marital Status |  |  |  |  |  |
| Married / life partners | 29 (52.8) |  |  | 12 (23.1) |  |
| Divorced | 8 (14.5) |  |  | 9 (17.3) |  |
| Single | 16 (29.1) |  |  | 22 (42.3) |  |
| Widowed | 2 (3.6) |  |  | 1 (1.9) |  |
| Employment status |  |  |  |  |  |
| Student / student worker | 7 (12.8) |  |  | 1 (1.9) |  |
| Employed full-time | 24 (43.6) |  |  | 11 (21.2) |  |
| Employed part-time | 2 (3.6) |  |  | 2 (3.8) |  |
| Unemployed | 11 (20.0) |  |  | 26 (50.0) |  |
| Retired | 11 (20.0) |  |  | 3 (5.8) |  |
| Number of children |  |  |  |  |  |
| 0 | 16 (29.1) |  |  | 17 (32.7) |  |
| 1 | 12 (21.8) |  |  | 13 (25.0) |  |
| 2 | 20 (36.4) |  |  | 10 (19.2) |  |
| 3 | 5 (9.1) |  |  | 2 (3.8) |  |
| 4 | 1 (1.8) |  |  | 2 (3.8) |  |
| Prior Psychological Treatment (No) | 24 (43.6) |  |  | n/a |  |
| Psychotropic Medication (No) | 15 (27.3) |  |  | n/a |  |
| Psychiatric Diagnosis |  |  |  |  |  |
| Depression/Anxiety | 18 (32.7) |  |  | n/a |  |
| Substance misuse | 1 (1.8) |  |  | n/a |  |
| Unknown | 36 (65.5) |  |  | n/a |  |
| First addiction treatment (No) | n/a |  |  | 14 (26.9) |  |

SUPLEMENTARY Table 2. Matching between PSYCHLOPS sub-themes and CORE-OM items.

| PSYCHLOPS Sub-theme | CORE-OM items | | | | Free text PSYCHLOPS response (e.g.) |
| --- | --- | --- | --- | --- | --- |
|  | Definite Yes | Possible  Yes | Possible No | No |  |
| Work-related problems |  |  |  | All 34 CORE-OM items. | “*The fact that I cannot achieve the goals at work”* |
| Sexual problems |  |  |  | All 34 CORE-OM items. | *“Having sexual intercourse with my wife”* |
| Another person’s illness |  |  |  | All 34 CORE-OM items. |  |
| Understanding self/events |  |  |  | All 34 CORE-OM items |  |
| Making decisions |  |  |  | All 34 CORE-OM items | *“It is hard for me to make decisions for the future”* |
| Relationship difficulties: family – worry about another |  |  |  | All 34 CORE-OM items. | *“The problem that worries me most is the disease of my mother…”* |
| Money worries |  |  |  | All 34 CORE-OM items. | *“Not enough money”* |
| Going out/traveling |  |  |  | All 34 CORE-OM items. | *“Travel”* |
| Having time |  |  |  | All 34 CORE-OM items. | *“I lose a lot of time”* |
| Housing worries |  |  |  | All 34 CORE-OM items. | *“Housekeeping”* |
| Avoiding issues |  |  |  | All 34 CORE-OM items. | *“Go search my personal things”* |
| Eating problems |  |  |  | All 34 CORE-OM items. | *“Eat”* |
| Existence/existential |  |  |  | All 34 CORE-OM items | *“The feel that most of the time it's not me…”* |
| Global |  |  |  | All 34 CORE-OM items | *“My well-being, my life in general.”* |
| Relationship difficulties: partner – worry about another |  |  |  | All 34 CORE-OM items. | *“My husband’s problem”* |
| Academic-related problems |  |  |  | All 34 CORE-OM items. | *“The studies”* |
| Justice-related problems |  |  |  | All 34 CORE-OM items. | *“My problem with justice”* |
| Sleep problems | 18 |  |  | 1; 2; 3; 4; 5; 6; 7; 8; 9; 10; 11; 12; 13; 14; 15; 16; 17; 19; 20; 21; 22; 23; 24; 25; 26; 27; 28; 29; 30; 31; 32; 33; 34. | *“Sleep badly”* |
| Aggression/irritability | 6; 22; 29; 34. |  |  | 1; 2; 3; 4; 5; 7; 8; 9; 10; 11; 12; 13; 14; 15; 16; 17; 18; 19; 20; 21; 23; 24; 25; 26; 27; 28; 30; 31; 32; 33. | *“Keep me calm and do things calmly and without stress”* |
| Future | 31 |  |  | 1; 2; 3; 4; 5; 6; 7; 8; 9; 10; 11; 12; 13; 14; 15; 16; 17; 18; 19; 20; 21; 22; 23; 24; 25; 26; 27; 28; 29; 30; 32; 33; 34. | *“I'm not sure of my professional future”* |
| Communication | 10 |  |  | 1; 2; 3; 4; 5; 6; 7; 8; 9; 11; 12; 13; 14; 15; 16; 17; 18; 19; 20; 21; 22; 23; 24; 25; 26; 27; 28; 29; 30; 31; 32; 33; 34. | *“Communicate”* |
| Guilt | 30 |  |  | 1; 2; 3; 4; 5; 6; 7; 8; 9; 10; 11; 12; 13; 14; 15; 16; 17; 18; 19; 20; 21; 22; 23; 24; 25; 26; 27; 28; 29; 31; 32; 33; 34. | *“Following an abortion”* |
| Outlook on life | 12 |  |  | 1; 2; 3; 4; 5; 6; 7; 8; 9; 10; 11; 13; 14; 15; 16; 17; 18; 19; 20; 21; 22; 23; 24; 25; 26; 27; 28; 29; 30; 31; 32; 33; 34. |  |
| Suicidal thoughts | 9; 16; 24. |  |  | 1; 2; 3; 4; 5; 6; 7; 8;10; 11; 12; 13; 14; 15; 17; 18; 19; 20; 21; 22; 23; 25; 26; 27; 28; 29; 30; 31; 32; 33; 34. |  |
| Traumatic event |  | 13; 28 |  | 1; 2; 3; 4; 5; 6; 7; 8; 9; 10; 11; 12; 14; 15; 16; 17; 18; 19; 20; 21; 22; 23; 24; 25; 26; 27; 29; 30; 31; 32; 33; 34. |  |
| Emotions - unspecified |  | 2; 14; 27. |  | 1; 3; 4; 5; 6; 7; 8; 9; 10; 11; 12; 13; 15; 16; 17; 18; 19; 20; 21; 22; 23; 24; 25; 26; 28; 29; 30; 31; 32; 33; 34. | *“Things around me affect me a lot and make me down”* |
| Dependence on other people |  | 3 |  | 1; 2; 4; 5; 6; 7; 8; 9; 10; 11; 12; 13; 14; 15; 16; 17; 18; 19; 20; 21; 22; 23; 24; 25; 26; 27; 28; 29; 30; 31; 32; 33; 34. | *“The fact that I had never lived alone, at my own risk”* |
| Coping: feelings |  | 13 |  | 1; 2; 3; 4; 5; 6; 7; 8; 9; 10; 11; 12; 14; 15; 16; 17; 18; 19; 20; 21; 22; 23; 24; 25; 26; 27; 28; 29; 30; 31; 32; 33; 34. |  |
| OCD (Obsessive-compulsive disorder) |  | 2; 11; 13; 28. |  | 1; 3; 4; 5; 6; 7; 8; 9; 10; 12; 14; 15; 16; 17; 18; 19; 20; 21; 22; 23; 24; 25; 26; 27; 29; 30; 31; 32; 33; 34. |  |
| Addiction |  |  | 13; 28. | 1; 2; 3; 4; 5; 6; 7; 8; 9; 10; 11; 12; 14; 15; 16; 17; 18; 19; 20; 21; 22; 23; 24; 25; 26; 27; 29; 30; 31; 32; 33; 34. | *“Drug Addiction problem”* |
| Personal development |  |  | 4; 32 | 1; 2; 3; 5; 6; 7; 8; 9; 10; 11; 12; 13; 14; 15; 16; 17; 18; 19; 20; 21; 22; 23; 24; 25; 26; 27; 28; 29; 30; 31; 33; 34. | *“The lack of emotional stability…”* |
| Relationship difficulties: family – caring |  |  | 3; 19. | 1; 2; 4; 5; 6; 7; 8; 9; 10; 11; 12; 13; 14; 15; 16; 17; 18; 20; 21; 22; 23; 24; 25; 26; 27; 28; 29; 30; 31; 32; 33; 34. |  |
| Thinking rationally |  |  | 13 | 1; 2; 3; 4; 5; 6; 7; 8; 9; 10; 11; 12; 14; 15; 16; 17; 18; 19; 20; 21; 22; 23; 24; 25; 26; 27; 28; 29; 30; 31; 32; 33; 34. |  |
| Concentration |  |  | 5; 11; 21. | 1; 2; 3; 4; 6; 7; 8; 9; 10; 12; 13; 14; 15; 16; 17; 18; 19; 20; 22; 23; 24; 25; 26; 27; 28; 29; 30; 31; 32; 33; 34. | *“Concentration at work”* |
| Moving on | 20 | 7; 17; 21; 23; 31 |  | 1; 2; 3; 4; 5; 6; 8; 9; 10; 11; 12; 13; 14; 15; 16; 18; 19; 22; 24; 25; 26; 27; 28; 29; 30; 32; 33; 34. | *“Walking forward with life”* |
| Coping: general | 7; 20. | 17 |  | 1; 2; 3; 4; 5; 6; 8; 9; 10; 11; 12; 13; 14; 15; 16; 18; 19; 21; 22; 23; 24; 25; 26; 27; 28; 29; 30; 31; 32; 33; 34. | *“Know how I will respond when faced with this kind of problem…”* |
| Loneliness/being alone | 1; 3; 26. | 19 |  | 2; 4; 5; 6; 7; 8; 9; 10; 11; 12; 13; 14; 15; 16; 17; 18; 20; 21; 22; 23; 24; 25; 27; 28; 29; 30; 31; 32; 33; 34. | *“Being alone”* |
| Having positive outlook | 31 | 12; 32 |  | 1; 2; 3; 4; 5; 6; 7; 8; 9; 10; 11; 13; 14; 15; 16; 17; 18; 19; 20; 21; 22; 23; 24; 25; 26; 27; 28; 29; 30; 33; 34. | *“Be positive”* |
| Somatic symptoms | 8 | 2; 14. |  | 1; 3; 4; 5; 6; 7; 9; 10; 11; 12; 13; 15; 16; 17; 18; 19; 20; 21; 22; 23; 24; 25; 26; 27; 28; 29; 30; 31; 32; 33; 34. | *“Constant physical pain”* |
| Motivation | 5 | 21; 32 |  | 1; 2; 3; 4; 6; 7; 8; 9; 10; 11; 12; 13; 14; 15; 16; 17; 18; 19; 20; 22; 23; 24; 25; 26; 27; 28; 29; 30; 31; 33; 34. | *“Unmotivated in my home”* |
| Coping: daily living | 7; 17; 20 | 11; 23. |  | 1; 2; 3; 4; 5; 6; 8; 9; 10; 12; 13; 14; 15; 16; 18; 19; 21; 22; 24; 25; 26; 27; 28; 29; 30; 31; 32; 33; 34. | *“I’m not able to do the normal life…”* |
| Attempted suicide | 34 | 9; 16; 24. |  | 1; 2; 3; 4; 5; 6; 7; 8; 10; 11; 12; 13; 14; 15; 17; 18; 19; 20; 21; 22; 23; 25; 26; 27; 28; 29; 30; 31; 32; 33. | *“I tried to kill myself”* |
| Self-harm | 34 | 9; 16; 24. |  | 1; 2; 3; 4; 5; 6; 7; 8; 10; 11; 12; 13; 14; 15; 17; 18; 19; 20; 21; 22; 23; 25; 26; 27; 28; 29; 30; 31; 32; 33. | *“Cut myself”* |
| Self-acceptance | 4 |  | 9 | 1; 2; 3; 5; 6; 7; 8; 10; 11; 12; 13; 14; 15; 16; 17; 18; 19; 20; 21; 22; 23; 24; 25; 26; 27; 28; 29; 30; 31; 32; 33; 34. |  |
| Relationship difficulties: partner – conflict |  | 1; 3; 10; 19; 25; 33. | 6; 22; 29. | 2; 4; 5; 7; 8; 9; 11; 12; 13; 14; 15; 16; 17; 18; 20; 21; 23; 24; 26; 27; 28; 30; 31; 32; 34. | *“It is difficult to live and talk to my girlfriend…”* |
| Relationship difficulties: family – breaking up |  | 1 | 3; 10; 14; 19; 27. | 2; 4; 5; 6; 7; 8; 9; 11; 12; 13; 15; 16; 17; 18; 20; 21; 22; 23; 24; 25; 26; 28; 29; 30; 31; 32; 33; 34. |  |
| Victim of abuse/sexual violence |  | 13; 28; 33. | 15 | 1; 2; 3; 4; 5; 6; 7; 8; 9; 10; 11; 12; 14; 16; 17; 18; 19; 20; 21; 22; 23; 24; 25; 26; 27; 29; 30; 31; 32; 34. | *“Psychological violence”* |
| Relationship difficulties: family – development |  | 19 | 1; 3; 27; 31. | 2; 4; 5; 6; 7; 8; 9; 10; 11; 12; 13; 14; 15; 16; 17; 18; 20; 21; 22; 23; 24; 25; 26; 28; 29; 30; 32; 33; 34. | *“Not having family”* |
| Relationship difficulties: partner – forming |  | 19 | 1; 3; 27; 31. | 2; 4; 5; 6; 7; 8; 9; 10; 11; 12; 13; 14; 15; 16; 17; 18; 20; 21; 22; 23; 24; 25; 26; 28; 29; 30; 32; 33; 34. |  |
| Thoughts |  | 13 | 9; 24 | 1; 2; 3; 4; 5; 6; 7; 8; 10; 11; 12; 14; 15; 16; 17; 18; 19; 20; 21; 22; 23; 25; 26; 27; 28; 29; 30; 31; 32; 33; 34. | *“My way of thinking, my negativity "not seeing anything positive…"* |
| Bereavement |  | 14; 27. | 1; 31. | 2; 3; 4; 5; 6; 7; 8; 9; 10; 11; 12; 13; 15; 16; 17; 18; 19; 20; 21; 22; 23; 24; 25; 26; 28; 29; 30; 32; 33; 34. | *“The death of my father”.* |
| Relationship difficulties: family-general |  | 1; 3; 10; 19; 25; 33. | 6; 22; 29. | 2; 4; 5; 7; 8; 9; 11; 12; 13; 14; 15; 16; 17; 18; 20; 21; 23; 24; 26; 27; 28; 30; 31; 32; 34. | *“The family”* |
| Relationship difficulties: partner – breaking up |  | 1 | 3; 10; 14; 19; 27. | 2; 4; 5; 6; 7; 8; 9; 11; 12; 13; 15; 16; 17; 18; 20; 21; 22; 23; 24; 25; 26; 28; 29; 30; 31; 32; 33; 34. | *“The separation with my wife”* |
| Relationship difficulties: partner – development |  | 19 | 1; 3; 27; 31. | 2; 4; 5; 6; 7; 8; 9; 10; 11; 12; 13; 14; 15; 16; 17; 18; 20; 21; 22; 23; 24; 25; 26; 28; 29; 30; 32; 33; 34. |  |
| Relationship difficulties: partner – general |  | 19; 31. | 1; 3; 27. | 2; 4; 5; 6; 7; 8; 9; 10; 11; 12; 13; 14; 15; 16; 17; 18; 20; 21; 22; 23; 24; 25; 26; 28; 29; 30; 32; 33; 34. | *“The way to deal with my husband”* |
| Relationship difficulties: family – conflict |  | 1; 3; 10; 19; 25; 33. | 6; 22; 29. | 2; 4; 5; 7; 8; 9; 11; 12; 13; 14; 15; 16; 17; 18; 20; 21; 23; 24; 26; 27; 28; 30; 31; 32; 34. | *“My father tease me and my mother and anything is god to start a discussion”* |
| Depression/anxiety | 2; 11; 27 | 1; 4; 5; 9; 13; 14; 15; 16; 20; 23; 24; 31. | 17; 18; 21; 28; 34. | 6; 7; 8; 10; 12; 19; 22; 25; 26; 29; 30; 32; 33. | *“I felt nervous”.* |
| Self image/self worth | 4 | 7; 12; 21; 30; 33. | 17; 24; 25; 27; 32. | 1; 2; 3; 5; 6; 8; 9; 10; 11; 13; 14; 15; 16; 18; 19; 20; 22; 23; 26; 28; 29; 31; 34. | “*My lack of self-esteem and to what extent is influencing my children”.* |
| Achievement | 12; 21; 32. | 7; 11; 31. | 4; 5; 17. | 1; 2; 3; 6; 8; 9; 10; 13; 14; 15; 16; 18; 19; 20; 22; 23; 24; 25; 26; 27; 28; 29; 30; 33; 34. | *“Not having my own income”* |
| Relationships – general | 10; 26. | 1; 3; 19; 25; 29; 33. | 6; 22. | 2; 4; 5; 7; 8; 9; 11; 12; 13; 14; 15; 16; 17; 18; 20; 21; 23; 24; 27; 28; 30; 31; 32; 34. | *“Confront people”* |
| Fears/panics | 15. | 2; 11; 13. | 28. | 1; 3; 4; 5; 6; 7; 8; 9; 10; 12; 14; 16; 17; 18; 19; 20; 21; 22; 23; 24; 25; 26; 27; 29; 30; 31; 32; 33; 34. | “I'm afraid of not having strength to handle what I'm going”. |
| Relaxing | 2; 11. | 8 | 13; 15; 28. | 1; 3; 4; 5; 6; 7; 9; 10;12; 14; 16; 17; 18; 19; 20; 21; 22; 23; 24; 25; 26; 27; 29; 30; 31; 32; 33; 34. |  |
| Worries about health | 8 | 4 | 7; 14; 27; 34. | 1; 2; 3; 5; 6; 9; 10; 11; 12; 13; 15; 16; 17; 18; 19; 20; 21; 22; 23; 24; 25; 26; 28; 29; 30; 31; 32; 33. | *“My health”.* |
| Being happy | 4; 27 | 12; 14; 19; 26; 33. | 1; 5; 20. | 2; 3; 6; 7; 8; 9; 10; 11; 13; 15; 16; 17; 18; 21; 22; 23; 24; 25; 28; 29; 30; 31; 32; 34. | “*Cannot feel joy for the normal things, for the children for example”* |
| Socialising | 1; 10; 26. | 3; 29. | 6; 22; 25; 33. | 2; 4; 5; 7; 8; 9; 11; 12; 13; 14; 15; 16; 17; 18; 19; 20; 21; 23; 24; 27; 28; 30; 31; 32; 34. | *“Having a night out. Going to a dinner. Without problems”* |

Note. The numbers on this table correspond to CORE-OM items: 1. I have felt terribly alone and isolated; 2. I have felt tense, anxious or nervous; 3. I have felt I have someone to turn to for support when needed; 4. I have felt O.K. about myself; 5. I have felt totally lacking in energy and enthusiasm; 6. I have been physically violent to others; 7. I have felt able to cope when things go wrong; 8. I have been troubled by aches, pains or other physical problems; 9. I have thought of hurting myself; 10. Talking to people has felt too much for me; 11. Tension and anxiety have prevented me doing important things; 12. I have been happy with the things I have done; 13. I have been disturbed by unwanted thoughts and feelings; 14. I have felt like crying; 15. I have felt panic or terror; 16. I made plans to end my life; 17. I have felt overwhelmed by my problems; 18. I have difficulty getting to sleep or staying asleep; 19. I have felt warmth and affection for someone; 20. My problems have been impossible to put to one side; 21. I have been able to do most things I needed to; 22. I have threatened or intimidated another person; 23. I have felt despairing or hopeless; 24. I have thought it would be better if I were dead; 25. I have felt criticized by other people; 26. I have thought I have no friends; 27. I have felt unhappy; 28. Unwanted images or memories have been distressing me; 29. I have been irritable when with other people; 30. I have thought I am to blame for my problems and difficulties; 31. I have felt optimistic about my future; 32. I have achieved the things I wanted to; 33. I have felt humiliated or shamed by other people; 34. I have hurt myself physically or taken dangerous risks with my health.

SUPLEMENTARY Table 3. Matching between PSYCHLOPS sub-themes and PHQ-9 items.

| PSYCHLOPS Sub-theme | PHQ-9 items | | | | Free text PSYCHLOPS response (e.g.) |
| --- | --- | --- | --- | --- | --- |
|  | Yes | Possible Yes | Possible No | No |  |
| Work-related problems |  |  |  | All PHQ-9 items. | “*The fact that I cannot achieve the goals at work”* |
| Moving on |  |  |  | All PHQ-9 items. | *“Walking forward with life”* |
| Relationships – general |  |  |  | All PHQ-9 items. | *“Confront people”* |
| Fears/panics |  |  |  | All PHQ-9 items. | *“I'm afraid of not having strength to handle what I'm going”* |
| Relationship difficulties: family-general |  |  |  | All PHQ-9 items | *“The family”* |
| Aggression/irritability |  |  |  | All PHQ-9 items. | *“Keep me calm and do things calmly and without stress”* |
| Relationship difficulties: partner – breaking up |  |  |  | All PHQ-9 items | *“The separation with my wife”* |
| Coping: general |  |  |  | All PHQ-9 items | *“Know how I will respond when faced with this kind of problem…”* |
| Relationship difficulties: partner – development |  |  |  | All PHQ-9 items. |  |
| Relationship difficulties: partner – general |  |  |  | All PHQ-9 items | *“The way to deal with my husband”* |
| Worries about health |  |  |  | All PHQ-9 items | *“My health”.* |
| Being happy |  |  |  | All PHQ-9 items. | “*Cannot feel joy for the normal things, for the children for example”* |
| Socialising |  |  |  | All PHQ-9 items. | *“Having a night out. Going to a dinner. Without problems”* |
| Loneliness/being alone |  |  |  | All PHQ-9 items. | *“Being alone”* |
| Sexual problems |  |  |  | All PHQ-9 items | *“Having sexual intercourse with my wife”* |
| Traumatic event |  |  |  | All PHQ-9 items. |  |
| Relationship difficulties: family – conflict |  |  |  | All PHQ-9 items. | *“My father tease me and my mother and anything is god to start a discussion”* |
| Relationship difficulties: partner – conflict |  |  |  | All PHQ-9 items. | *“It is difficult to live and talk to my girlfriend…”* |
| Addiction |  |  |  | All PHQ-9 items. | *“Drug Addiction problem”* |
| Another person’s illness |  |  |  | All PHQ-9 items. |  |
| Relationship difficulties: family – breaking up |  |  |  | All PHQ-9 items. |  |
| Victim of abuse/sexual violence |  |  |  | All PHQ-9 items. | *“Psychological violence”* |
| Coping: daily living |  |  |  | All PHQ-9 items. | *“I’m not able to do the normal life…”* |
| Money worries |  |  |  | All PHQ-9 items. | *“Not enough money”* |
| Relationship difficulties: family – development |  |  |  | All PHQ-9 items. | *“Not having family”* |
| Understanding self/events |  |  |  | All PHQ-9 items. |  |
| Making decisions |  |  |  | All PHQ-9 items. | *“It is hard for me to make decisions for the future”* |
| Relationship difficulties: family – worry about another |  |  |  | All PHQ-9 items. | *“The problem that worries me most is the disease of my mother…”* |
| Going out/traveling |  |  |  | All PHQ-9 items. | *“Travel”* |
| Guilt |  |  |  | All PHQ-9 items. | *“Following an abortion”* |
| Dependence on other people |  |  |  | All PHQ-9 items. | *“The fact that I had never lived alone, at my own risk”* |
| Having time |  |  |  | All PHQ-9 items. | *“I lose a lot of time”* |
| Housing worries |  |  |  | All PHQ-9 items. | *“Housekeeping”* |
| OCD (Obsessive-compulsive disorder) |  |  |  | All PHQ-9 items. |  |
| Relationship difficulties: partner – forming |  |  |  | All PHQ-9 items. |  |
| Avoiding issues |  |  |  | All PHQ-9 items. | *“Go search my personal things”* |
| Coping: feelings |  |  |  | All PHQ-9 items. |  |
| Existence/existential |  |  |  | All PHQ-9 items. | *“The feel that most of the time it's not me…”* |
| Global |  |  |  | All PHQ-9 items. | *“My well-being, my life in general.”* |
| Relationship difficulties: family – caring |  |  |  | All PHQ-9 items. |  |
| Relationship difficulties: partner – worry about another |  |  |  | All PHQ-9 items. | *“My husband’s problem”* |
| Academic-related problems |  |  |  | All PHQ-9 items. | *“The studies”* |
| Justice-related problems |  |  |  | All PHQ-9 items. | *“My problem with justice”* |
| Self image/self worth | 6 |  |  | 1; 2; 3; 4; 5; 7; 8; 9. | “*My lack of self-esteem and to what extent is influencing my children”* |
| Concentration | 7 |  |  | 1; 2; 3; 4; 5; 6; 8; 9. | *“Concentration at work”* |
| Sleep problems | 3 |  |  | 1; 2; 4; 5; 6; 7; 8; 9. | *“Sleep badly”* |
| Self-acceptance | 6 |  |  | 1; 2; 3; 4; 5; 7; 8; 9. |  |
| Eating problems | 5 |  |  | 1; 2; 3; 4; 6; 7; 8; 9. | *“Eat”* |
| Suicidal thoughts | 9 |  |  | 1; 2; 3; 4; 5; 6; 7; 8. |  |
| Future |  | 2 |  | 1; 3; 4; 5; 6; 7; 8; 9. | *“I'm not sure of my professional future”* |
| Having positive outlook |  | 2 |  | 1; 3; 4; 5; 6; 7; 8; 9. | *“Be positive”* |
| Communication |  | 8 |  | 1; 2; 3; 4; 5; 6; 7; 9 | *“Communicate”* |
| Emotions – unspecified |  | 6 |  | 1; 2; 3; 4; 5; 7; 8; 9 | *“Things around me affect me a lot and make me down”* |
| Outlook on life |  | 2 |  | 1; 3; 4; 5; 6; 7; 8; 9 |  |
| Personal development |  | 6 |  | 1; 2; 3; 4; 5; 7; 8; 9. | *“The lack of emotional stability…”* |
| Thoughts |  | 9 |  | 1; 2; 3; 4; 5; 6; 7; 8 | *“My way of thinking, my negativity "not seeing anything positive…"* |
| Achievement |  |  | 2 | 1; 3; 4; 5; 6; 7; 8; 9. | *“Not having my own income”* |
| Bereavement |  |  | 2 | 1; 3; 4; 5; 6; 7; 8; 9. | *“The death of my father”* |
| Relaxing |  |  | 8 | 1; 2; 3; 4; 5; 6; 7; 9. |  |
| Thinking rationally |  |  | 9 | 1; 2; 3; 4; 5; 6; 7; 8. |  |
| Attempted suicide |  |  | 9 | 1; 2; 3; 4; 5; 6; 7; 8 | *“I tried to kill myself”* |
| Self-harm |  |  | 9 | 1; 2; 3; 4; 5; 6; 7; 8 | *“Cut myself”* |
| Somatic symptoms | 4 | 8 |  | 1; 2; 3; 5; 6; 7; 9. | *“Constant physical pain”* |
| Motivation | 1 | 2 |  | 1; 3; 4; 5; 6; 7; 8; 9. | *“Unmotivated in my home”* |
| Depression/anxiety | 2 | 1; 6; 9 | 3; 4; 5; 7; 8. |  | *“I felt nervous”* |

Note. The numbers on this table correspond to PHQ-9 items: 1. Little interest or pleasure in doing things; 2. Felling down, depressed, or hopeless; 3. Trouble falling or staying asleep, or sleeping to much; 4. Feeling tired or having little energy; 5. Poor appetite or overeating; 6. Feeling bad about yourself – or that you are a failure or have let yourself or your family down; 7. Trouble concentrating on things, such as reading the newspaper or watching television; 8. Moving or speaking so slowly that other people could have noticed? Or the opposite – being so fidgety or restless that you have been moving around a lot more than usual; 9. Thoughts that you would be better off dead or of hurting yourself in some way.

SUPLEMENTARY Table 4. Summary of 65 sub-themes on PSYCHLOPS, the relative frequency of each sub-theme found, the frequency of patients that indicated each sub-theme and the matching with CORE-OM and PHQ-9.

| PSYCHLOPS Sub-themes | Frequency (%) of responses on PSYCHLOPS | | | Number (%) of patients making each PSYCHLOPS response | | | | | Matching with CORE-OM | | MatchPHQ-9 | |
| --- | --- | --- | --- | --- | --- | --- | --- | --- | --- | --- | --- | --- |
|  | Sample 1  (n=153 items) | Sample 2  (n=126 items) | Total Sample (n=279) | Sample 1  (n=55) | Sample 2  (n=52) | | Total sample (n=107) | |  | |  | |
| Work-related problems | 12 (8) | 19 (15) | 31(11) | 11 (20) | 17 (33) | | 28 (26) | | No | | No | |
| Relationship difficulties: family – worry about another | 17 (11) | 8 (6) | 25 (9) | 17 (31) | 8 (15) | | 25 (23) | | No | | No | |
| Addiction | 0 (0) | 22 (17) | 22 (8) | 0 (0) | 20 (38) | | 20 (19) | | Possible no | | No | |
| Money worries | 5 (3) | 12 (9) | 17 (6) | 5 (9) | 10 (19) | | 15 (14) | | No | | No | |
| Worries about health | 12 (8) | 2 (2) | 14 (5) | 8 (15) | 2 (4) | | 10 (9) | | Yes | | No | |
| Fears/Panics | 7 (5) | 1 (1) | 8 (3) | 7 (13) | 1 (2) | | 8 (7) | | Yes | | No | |
| Socialising | 7 (5) | 4 (3) | 11 (4) | 7 (13) | 4 (8) | | 11 (10) | | Yes | | No | |
| Relationship difficulties: family – general | 4 (3) | 5 (4) | 9 (3) | 4 (7) | 5 (10) | | 9 (8) | | Possible yes | | No | |
| Moving on | 6 (4) | 2 (2) | 8 (3) | 6 (11) | 2 (4) | | 8 (7) | | Yes | | No | |
| Loneliness/ being alone | 6 (4) | 2 (2) | 8 (3) | 6 (11) | 2 (4) | | 8 (7) | | Yes | | No | |
| Global | 5 (3) | 3 (2) | 8 (3) | 5 (9) | 3 (6) | | 8 (7) | | No | | No | |
| Relationships-general | 4 (3) | 3 (2) | 7 (3) | 4 (7) | 3 (6) | | 7 (7) | | Yes | | No | |
| Housing worries | 3 (2) | 4 (3) | 7 (3) | 3 (5) | 4 (8) | | 7 (7) | | No | | No | |
| Self image/self worth | 3 (2) | 3 (2) | 6 (2) | 3 (5) | 3 (6) | | 6 (6) | | Yes | | Yes | |
| Future | 4 (3) | 2 (2) | 6 (2) | 4 (7) | 2 (4) | | 6 (6) | | Yes | | Possible yes | |
| Coping: daily living | 3 (2) | 3 (2) | 6 (2) | 3 (5) | 3 (6) | | 6 (6) | | Yes | | No | |
| Depression/anxiety | 7 (5) | 0 (0) | 7 (3) | 6 (11) | 0 (0) | | 6 (6) | | Yes | | Yes | |
| Relationship difficulties: family – conflict | 3 (2) | 2 (2) | 5 (2) | 3 (5) | 2 (4) | | 5 (5) | | Possible yes | | No | |
| Relationship difficulties: partner – breaking up | 2 (1) | 3 (2) | 5 (2) | 2 (4) | 3 (6) | | 5 (5) | | Possible yes | | No | |
| Somatic symptoms | 5 (3) | 0 (0) | 5 (2) | 5 (9) | 0 (0) | | 5 (5) | | Yes | | Yes | |
| Bereavement | 2 (1) | 2 (1) | 4 (1) | 2 (4) | 1 (2) | | 3 (3) | | Possible yes | | Possible no | |
| Sleep problems | 3 (2) | 0 (0) | 3 (1) | 3 (5) | 0 (0) | | 3 (3) | | Yes | | Yes | |
| Relationship difficulties: partner – general | 3 (2) | 0 (0) | 3 (1) | 3 (5) | 0 (0) | | 3 (3) | | Possible yes | | No | |
| Motivation | 0 (0) | 3 (2) | 3 (1) | 0 (0) | 3 (6) | | 3 (3) | | Yes | | Yes | |
| Communication | 0 (0) | 3 (2) | 3 (1) | 0 (0) | 3 (6) | | 3 (3) | | Yes | | Possible yes | |
| Emotions – unspecified | 1 (1) | 2 (2) | 3 (1) | 1 (2) | 2 (4) | | 3 (3) | | Possible yes | | Possible yes | |
| Relationship difficulties: partner – worry about another | 3 (2) | 0 (0) | 3 (1) | 3 (5) | 0 (0) | | 3 (3) | | No | | No | |
| Justice-related problems | 0 (0) | 3 (2) | 3 (1) | 0 (0) | 3 (6) | | 3 (3) | | No | | No | |
| Achievement | 2 (1) | 1 (1) | 3 (1) | 2 (4) | 1 (2) | | 3 (3) | | Yes | | Possible no | |
| Sexual problems | 3 (2) | 0 (0) | 3 (1) | 2 (4) | 0 (0) | | 2 (2) | | No | | No | |
| Eating problems | 2 (1) | 1 (1) | 3 (1) | 1 (2) | 1 (2) | | 2 (2) | | No | | Yes | |
| Aggression/irritability | 0 (0) | 2 (2) | 2 (1) | 0 (0) | 2 (4) | | 2 (2) | | Yes | | No | |
| Relationship difficulties: family – development | 1 (1) | 1 (1) | 2 (1) | 1 (2) | 1 (2) | | 2 (2) | | Possible yes | | No | |
| Dependence on other people | 1 (1) | 1 (1) | 2 (1) | 1 (2) | 1 (2) | | 2 (2) | | Possible yes | | No | |
| Avoiding issues | 1 (1) | 1 (1) | 2 (1) | 1 (2) | 1 (2) | | 2 (2) | | No | | No | |
| Personal development | 2 (1) | 0 (0) | 2 (1) | 2 (4) | 0 (0) | | 2 (2) | | Possible no | | Possible yes | |
| Attempted suicide | 2 (1) | 0 (0) | 2 (1) | 2 (4) | 0 (0) | | 2 (2) | | Yes | | Possible no | |
| Self-harm | 2 (1) | 0 (0) | 2 (1) | 2 (4) | 0 (0) | | 2 (2) | | Yes | | Possible no | |
| Academic-related problems | 1 (1) | 1 (1) | 2 (1) | 1 (2) | 1 (2) | | 2 (2) | | No | | No | |
| Being happy | 1 (1) | 1 (1) | 2 (1) | 1 (2) | 1 (2) | | 2 (2) | | Yes | | No | |
| Relationship difficulties: partner – conflict | 2 (1) | 0 (0) | 2 (1) | 1 (2) | 0 (0) | | 1 (1) | | Possible yes | | No | |
| Concentration | 1 (1) | 0 (0) | 1 (0) | 1 (2) | 0 (0) | | 1 (1) | | Possible no | | Yes | |
| Coping: general | 1 (1) | 0 (0) | 1 (0) | 1 (2) | 0 (0) | | 1 (1) | | Yes | | No | |
| Having positive outlook | 0 (0) | 1 (1) | 1 (0) | 0 (0) | 1 (2) | | 1 (1) | | Yes | | Possible yes | |
| Victim of abuse/sexual violence | 1 (1) | 0 (0) | 1 (0) | 1 (2) | 0 (0) | | 1 (1) | | No | | No | |
| Making decisions | 1 (1) | 0 (0) | 1 (0) | 1 (2) | 0 (0) | | 1 (1) | | No | | No | |
| Going out/traveling | 0 (0) | 1 (1) | 1 (0) | 0 (0) | 1 (2) | | 1 (1) | | No | | No | |
| Guilt | 1 (1) | 0 (0) | 1 (0) | 1 (2) | 0 (0) | | 1 (1) | | Yes | | No | |
| Having time | 0 (0) | 1 (1) | 1 (0) | 0 (0) | 1 (2) | | 1 (1) | | No | | No | |
| Existence/existential | 0 (0) | 1 (1) | 1 (0) | 0 (0) | 1 (2) | | 1 (1) | | No | | No | |
| Thoughts | 1 (1) | 0 (0) | 1 (0) | 1 (2) | 0 (0) | | 1 (1) | | Possible yes | | Possible yes | |
|  |  |  |  |  |  | |  | |  | |  | |
|  | **Subthemes of the classification system not indicated by patients in our sample** | | | | | | | | | | | |
| Relationship difficulties: partner – development |  |  | 0 |  | |  | | 0 | | Possible yes | | No |
| Relaxing |  |  | 0 |  | |  | | 0 | | Yes | | Possible no |
| Traumatic event |  |  | 0 |  | |  | | 0 | | Possible yes | | No |
| Self-acceptance |  |  | 0 |  | |  | | 0 | | Yes | | Yes |
| Another person’s illness |  |  | 0 |  | |  | | 0 | | No | | No |
| Relationship difficulties: family – breaking up |  |  | 0 |  | |  | | 0 | | Possible yes | | No |
| Understanding self/events |  |  | 0 |  | |  | | 0 | | No | | No |
| Outlook on life |  |  | 0 |  | |  | | 0 | | Yes | | Possible yes |
| OCD (Obsessive-compulsive disorder) |  |  | 0 |  | |  | | 0 | | Possible yes | | No |
| Relationship difficulties: partner – forming |  |  | 0 |  | |  | | 0 | | Possible yes | | No |
| Coping: feelings |  |  | 0 |  | |  | | 0 | | Possible yes | | No |
| Relationship difficulties: family – caring |  |  | 0 |  | |  | | 0 | | Possible no | | No |
| Suicidal thoughts |  |  | 0 |  | |  | | 0 | | Yes | | Yes |
| Thinking rationally |  |  | 0 |  | |  | | 0 | | Possible no | | Possible no |

SUPLEMENTARY Table 5. Number (%) of patients in the clinical range that indicate free-text PSYCHLOPS items not covered by CORE-OM and PHQ-9 (with *definite no* matching themes).

|  | CORE-OM | | | PHQ-9 | | |
| --- | --- | --- | --- | --- | --- | --- |
|  | Sample 1 | Sample 2 | Total sample | Sample 1 | Sample 2 | Total sample |
| All themes match | 17 (32) | 12 (25) | 29 (29) | 3 (8) | 0 (0) | 3 (5) |
| One *definite no* matching theme | 23 (43) | 25 (52) | 48 (48) | 7 (19) | 5 (20) | 12 (20) |
| Two *definite no* matching themes | 11 (21) | 8 (17) | 19 (19) | 11 (31) | 12 (48) | 23 (38) |
| Three *definite no* matching themes | 2 (4) | 3 (6) | 5 (5) | 15 (42) | 8 (32) | 23 (38) |
|  | 53 (100) | 48 (100) | 101 (100) | 36 (100) | 25 (100) | 61 (100) |
